# Antifungal Effect of (+)-Pinoresinol Isolated from *Sambucus williamsii*

**DOI:** 10.3390/molecules15053507

**Published:** 2010-05-14

**Authors:** Bomi Hwang, Juneyoung Lee, Qing-He Liu, Eun-Rhan Woo, Dong Gun Lee

**Affiliations:** 1 School of Life Sciences and Biotechnology, College of Natural Sciences, Kyungpook National University, 1370 Sankyuk-dong, Puk-ku, Daegu 702-701, Korea; E-Mails: hwangb1@knu.ac.kr (B.H.); juneyounglee@knu.ac.kr (J.L.); dglee222@knu.ac.kr (D.-G.L.); 2 College of Pharmacy, Chosun University, 375 Seosuk-dong, Dong-gu, Gwangju 501-759, Korea; E-Mails: qinghe1978@hotmail.com (Q.-H.L.); wooer@chosun.ac.kr (E.-R.W.)

**Keywords:** (+)-pinoresinol, lignan, antifungal compound, membrane-active mechanism, *Sambucus williamsii* HANCE

## Abstract

In this study, we investigated the antifungal activity and mechanism of action of (+)-pinoresinol, a biphenolic compound isolated from the herb *Sambucus williamsii*, used in traditional medicine. (++)-Pinoresinol displays potent antifungal properties without hemolytic effects on human erythrocytes. To understand the antifungal mechanism of (+)-pinoresinol, we conducted fluorescence experiments on the human pathogen *Candida albicans.* Fluorescence analysis using 1,6-diphenyl-1,3,5-hexatriene (DPH) indicated that the (+)-pinoresinol caused damage to the fungal plasma membrane. This result was confirmed by using rhodamine-labeled giant unilamellar vesicle (GUV) experiments. Therefore, the present study indicates that (+)-pinoresinol possesses fungicidal activities and therapeutic potential as an antifungal agent for the treatment of fungal infectious diseases in humans.

## 1. Introduction

**Figure 1:**
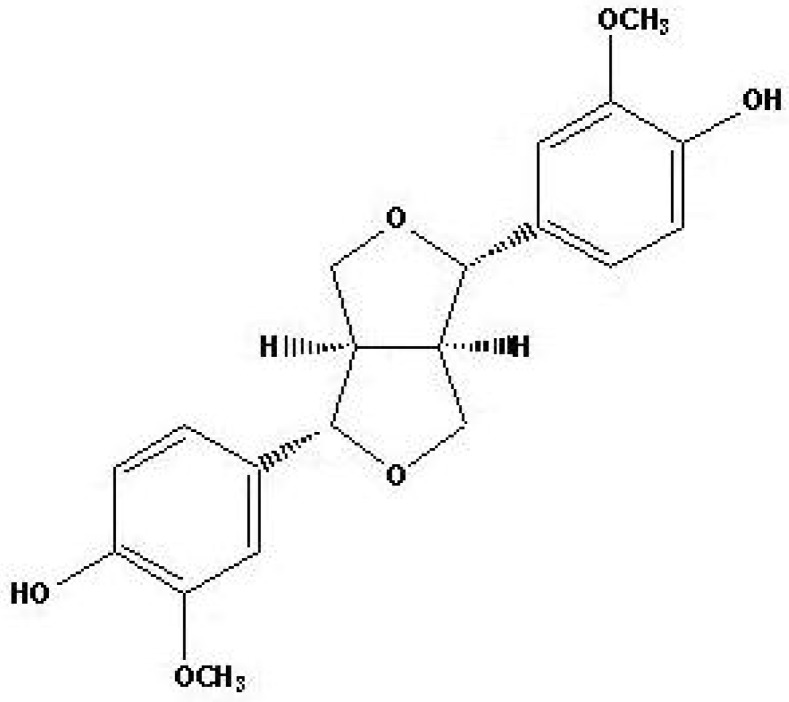
Chemical structure of (+)-pinoresinol derived from *Sambucus williamsii*.

The widespread use of classical antibiotics has resulted in the emergence of many drug-resistant strains of infectious pathogens. Consequently, numerous diseases are becoming increasingly difficult to treat as microorganisms responsible for severe infections in hospitalized patients, foodborne pathogens and sexually transmitted pathogens, have become resistant to most available antimicrobial drugs [1]. To effectively combat the grave situation created by these resistant microbial pathogens, and to decrease the morbidity and mortality caused by this resistance, the development of new classes of antibiotics is critical. Currently, natural products and their derivatives used in clinical surroundings represent more than 50% of all the drugs in the World. In particular, plants produce an armament of chemical compounds, generally known as secondary metabolites, which have the potential to protect plants from diseases caused by microbial invasion [2,3]. Therefore, there has been growing interest in the compounds derived from these plants as a source of alternative therapies and in the therapeutic use of natural products in general. 

(+)-Pinoresinol (Figure 1) is a biphenolic lignan isolated from the herb *Sambucus williamsii*, a folk medicinal plant used for its therapeutic properties. The genus *Sambucus*, widely distributed in Europe, Asia and North Africa, has been used in traditional medicine as an analgesic, antivirus, anti-inflammatory, homoeostatic, and diuretic drugs which act on bruises, fractures, and edema [4]. 

Lignans comprise a large class of secondary metabolites produced by oxidative dimerization of two phenylpropanoid units and have many biological properties, such as antioxidant, antitumor, antiviral, antibacterial, insecticidal, fungistatic and anti-platelet activities, in addition to protective effects against coronary heart disease [5,6]. Although (+)-pinoresinols are well-known for their various biological properties, not many studies have focused on their antifungal activity and its mechanism of action against opportunistic fungal pathogens. Therefore, in this study, we have investigated the antifungal activity and mechanism of action of (+)-pinoresinol and its suggested potential as therapeutic agents for fungal infections in humans.

## 2. Results and Discussion

### 2.1. Antifungal activity of (+)-pinoresinol

(+)-Pinoresinol, a biphenolic lignan of wide distribution in plants, was isolated from the herbal *Sambucus williamsii*. Amphotericin B, a fungicidal agent used to treat the most serious life-threatening fungal infections, was used as a positive control compound [7]. The mechanism of action of amphotericin B is based on the binding of ergosterol on the fungal cell membrane. The binding causes disorganization of the cellular membrane and an increase in fungal membrane permeability, eventually leading to cell death [8,9,10].

The antifungal effect of (+)-pinoresinol was measured by the 3-(4,5-dimethyl-2-thiazolyl)-2,5-diphenyl-2*H*-tetrazolium bromide (MTT) assay [20]. (+)-Pinoresinol, with MIC values of 12.5–25 μg/mL, exhibited antifungal activity against several human pathogenic fungi, including *Candida albicans*, *Trichosporon beigelii* and *Malassezia furfur* (Table 1).

These fungal strains exist in humans as commensals and are superficial contaminants that can be present in a variety of serious infections. Although this compound exhibited a less potent antifungal activity than that of amphotericin B, it was thought that the (+)-pinoresinol nevertheless displayed significant antifungal properties. Additionally, we confirmed the antifungal activity of (+)-pinoresinol by measuring the releases of trehalose. In yeast and bacteria, trehalose (α-D-glucopyranosyl-1,1-α-D-glucopyranoside) is accumulated because of environmental stresses such as desiccation, dehydration, oxidation, heat, freezing temperatures, and toxic agents [11]. The glucose concentration significantly increased in *C. albicans* cells incubated with (+)-pinoresinol as compared with (+)-pinoresinol-untreated cells. This increase in glucose concentration is thought to reveal an accumulation of intracellular trehalose caused by the antifungal activities of (+)-pinoresinol (Table 2).

**Figure 2:**
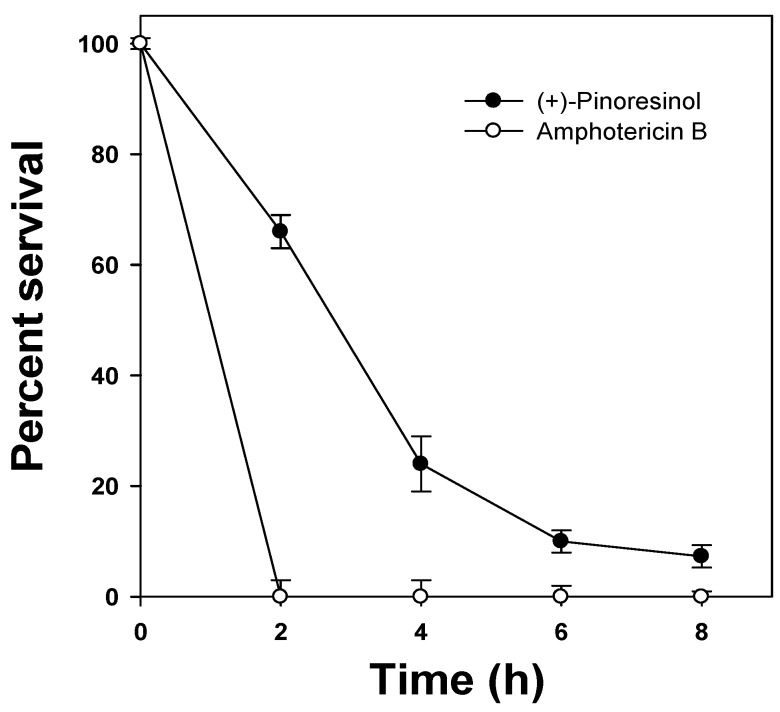
Time-killing plots for *C. albicans* by compounds. *C. albicans* cells were incubated with 25 μg/mL of (+)-pinoresinol or 12.5 μg/mL of amphotericin B. The viability was determined every 2 h by using colony forming units (CFUs) and expressed as a percentage of survivals, and the error bars represent the standard deviation (S.D.) values for three independent experiments, performed in triplicate.

These results suggested that (+)-pinoresinol can exert significant antifungal activity against human pathogenic fungal strains. Finally, to assess the antifungal activity and killing potency of (+)-pinoresinol, a time-killing assay was conducted by counting the colony-forming units (CFUs) of *C. albicans*. The CFUs of *C. albicans* cells under two times the minimum inhibitory concentration (MIC) of (+)-pinoresinol decreased similarly to the manner that CFUs rapidly decreased in the presence of amphotericin B (Figure 2). In summary, these results demonstrated that (+)-pinoresinol has antifungal activities against human fungal pathogens.

### 2.2. Hemolytic activity of (+)-pinoresinol

The cytotoxic properties of (+)-pinoresinol were determined by measuring its hemolytic activity against human erythrocytes. Amphotericin B acts on fungal cell membranes by binding to ergosterol and has the capability of binding to the cholesterol in mammalian cell membranes, which is associated with toxicity problems in humans [12]. As shown in Table 3, amphotericin B had significant hemolytic activity; however, (+)-pinoresinol exhibited no hemolytic activity at any concentration. This indicated that (+)-pinoresinol, might be used in clinical use for human diseases, without cytotoxicity.

### 2.3. Membrane-active mechanism(s) of (+)-pinoresinol

**Figure 3:**
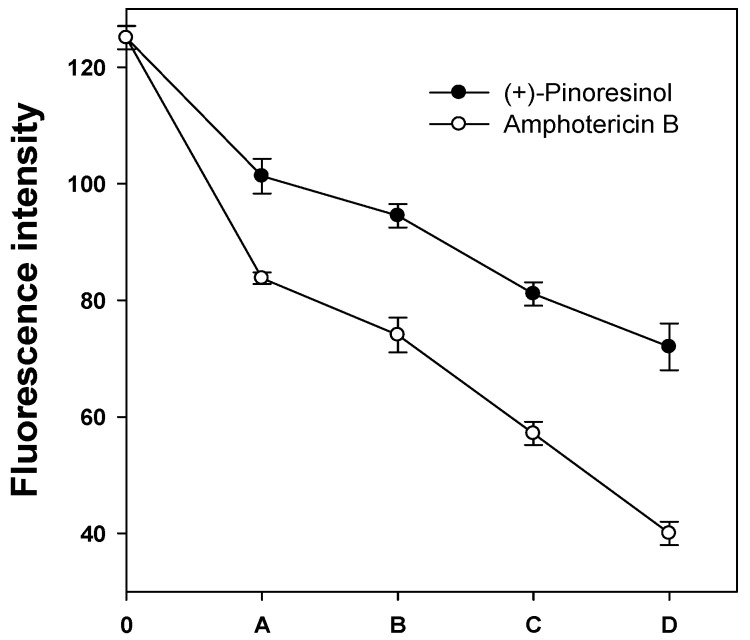
DPH fluorescence intensity after adding of compounds. Treatment with A, 6.25; B, 12.5; C, 25; D, 50 μg/mL of (+)-pinoresinol or treatment with A, 3.13; B, 6.25; C, 12.5; D, 25 μg/mL of amphotericin B. The error bar represents the standard deviation (S.D.) values for three independent experiments, performed in triplicate.

To provide information about the mode of antifungal action of (+)-pinoresinol, we selected *C. albicans*, which is the most prevalent human fungal pathogen and causes life-threatening systemic infections of superficial mucosal surfaces such as candidiasis [13]. To elucidate the *in vivo* membrane fluidity casued by (+)-pinoresinol in the plasma membrane of *C. albicans*, changes in the membrane dynamics were investigated by using 1,6-diphenyl-1,3,5-hexatriene (DPH) as a membrane probe. If the antifungal activities were exerted by (+)-pinoresinol at the level of the plasma membrane, DPH, which interacts with the lipophilic tail group of the phospholipids in the cytoplasmic membrane, could not disturb the structure of the bilayer membrane [14]. The plasma membrane DPH fluorescence intensity was decreased by increasing the concentrations of (+)-pinoresinol, but was less than the decrease observed in the presence of amphotericin B (Figure 3). The result indicates that (+)-pinoresinol exerts its fungicidal activities by perturbing the cytoplasmic membranes of *C. albicans*.

**Figure 4:**
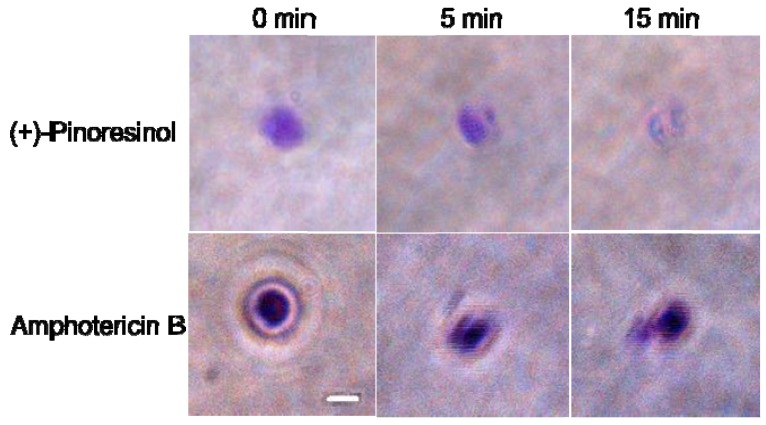
The response of the single GUV labeled with rhodamine to the treatment with compounds. The times above each image show the time after the addition of (+)-pinoresinol or amphotericin B. The scale bar corresponds to 10 μm.

In order to further evaluate and confirm the mechanisms of antifungal action of (+)-pinoresinol, fungal membrane mimetic liposomes were employed. Giant unilamellar vesicles (GUVs) with an average diameter ranging from 10 to 100 μm have been used for the observation of the physical and biological properties of models imitating vesicle membranes, such as macroscopic and morphological changes [15,16]. The GUVs are easily formed from different lipid mixtures and can be observed under a fluorescent or confocal microscope provided that the appropriate fluorescent probe is incorporated into the lipid phase during vesicle formation [17]. They have been used in the studies aimed at investigating the mechanisms of action of compounds or peptides. We used GUVs of artificial lipid bilayers mimicking the membrane of *C. albicans* [PC:Rho-PE:PI:ergosterol, 5:4:1:2 (w/w/w/w)][18]. In our experiments, (+)-pinoresinol caused circular shape changes and a progressive decrease of rhodamine intensity for single GUVs (Figure 4). This result suggested that (+)-pinoresinol might exert its activity by forming pores in fungal model membranes. This result may also indicate the membrane-active mechanism of (+)-pinoresinol.

## 3. Experimental

### 3.1. Extraction and isolation of compound from Sambucus williamsii

The stem bark of *Sambucus williamsii* was collected from the Herbarium of College of Pharmacy, Chosun University, Korea, in April 2003. A voucher specimen was deposited in the Herbarium of College of Pharmacy, Chosun University (CSU-994-17). The air-dried stem bark of *Sambucus williamsii* (840 g) was cut and extracted with MeOH under reflux. The methanol extract (57.1 g) was suspended in water and then partitioned sequentially with equal volumes of dichloromethane, ethyl acetate, and *n*-butanol. Each fraction was evaporated *in vacuo* to yield the corresponding CH_2_Cl_2_ (18.61 g), EtOAc (5.02 g), *n*-BuOH (1.96 g), and water (19.07 g) extract residues. The CH_2_Cl_2_ extract (4.2 g) was purified by column chromatography on a silica gel eluting with an *n*-hexane-EtOAc = 100:1→ 1:1, CHCl_3_-MeOH-H_2_O = 30:1:0.1 → 1:1:0.1 gradient system. The fractions were combined based on their TLC patterns to yield subfractions designated as D1-D6. Subfraction D6 (1.27 g) was further purified by column chromatography over silica gel eluting with an *n*-hexane-EtOAc = 100:1→ 1:1, CHCl_3_-MeOH-H2O = 30:1:0.1 → 1:1:0.1 gradient system to afford seven subfractions (D61-D67). Subfraction D63 (141.36 mg) was purified by repeated Sephadex LH-20 column chromatography (MeOH-H_2_O = 2:3) to give (+)-pinoresinol (46.58 mg). The physical and chemical data, including MS, ^1^H-NMR, ^13^C-NMR and HSQC of (+)-pinoresinol were identical with those reported previously [19,20,21].

### 3.2. Fungal strains and culture conditions

*T. beigelii* (KCTC 7707) and *M. furfur* (KCTC 7744) were obtained from the Korean Collection for Type Culture (KCTC) of the Korea Research Institute of Bioscience and Biotechnology (KRIBB), Daejeon, Korea. *C. albicans* (ATCC 90028) was obtained from the American Type Culture Collection (ATCC) (Manassas, VA, USA). *T. beigelii* and *C. albicans* were cultured in an YPD broth (Difco) with aeration at 28 °C and *M. furfur* was cultured at 32 °C in a modified Bacto yeast extract/malt extract (YM) broth (Difco) and 1% olive oil.

### 3.3. Determining of antifungal susceptibility

Fungal cells (1 × 10^8^ /mL) were inoculated into an YPD or YM broth and 0.1 mL/well were dispensed into 96-well microtiter plates. Two-fold diluted (+)-pinoresinol was added to each fungal cell. After 48 h of incubation at either 28 °C or 32 °C, 3-(4,5-dimethyl-2-thiazolyl)-2,5-diphenyl-2*H*-tetrazolium bromide (MTT) solution [5 mg/mL MTT in phosphate-buffered saline (PBS), pH 7.4 was added to each well [22]. The minimal concentration of (+)-pinoresinol to prevent the growth of a given test organism was determined and defined as MIC. Growth was assayed with a microtiter ELISA reader (Molecular Devices Emax, CA, USA) by monitoring absorption at 580 nm. MIC values were determined by three independent assays.

### 3.4. Determination of intracellular glucose and trehalose

*C. albicans* cells (1 × 10^8^ /mL) containing (+)-pinoresinol (25 μg/mL, at two times the MIC) or amphotericin B (12.5 μg/mL, at two times the MIC) were incubated for 2 h at 28 °C. Cells were settled by centrifugation at 13,000 rpm for 10 min. The pellets were dried to calculate their dry weight and supernatants were transferred to a new tube. Intracellular trehalose was extracted from 3 mg (dry weight) of fungal cells with 0.025 mM potassium-phosphate buffer (pH 6.6), and then crude neutral trehalose-containing extractions were extracted by removing the cell debris. Released glucose and trehalose-containing supernatants were added to 0.05 units of trehalase. After 1 h of enzymatic reaction at 37 °C, the reaction suspension was mixed with water and 16% DNS reagent (3, 5-Dinitrosalicylic acid 1%, NaOH 2%, Sodium potassium tartrate 20%) was added [23]. For the reaction of glucose with the DNS reagent, the mixture was boiled for 5 min and cooled. Level of color formation was measured with an OPTIZEN 2120UV spectrophotometer (Mecasys, Daejeon, Korea) at a wavelength of 525 nm. The resulted represent the average of the measurements conducted in triplicate of three independent assays.

### 3.5. Kinetics of fungal killing

Exponential phased *C. albicans* cells (2 × 10^4^ CFUs/mL) were incubated with (+)-pinoresinol (25 μg/mL, at two times the MIC) or amphotericin B (12.5 μg/mL, at two times the MIC) used as positive control. The cultures were obtained and spread on an YPD agar plate, and then the colony forming units (CFUs) were counted after 24 h incubation at 28 °C [24]. The values were the average of triplicate measurements in three independent assays.

### 3.6. Hemolytic activity assay

The hemolytic activity of compound was assayed by determining the hemoglobin leakage from a 4% suspension of fresh human erythrocytes at 414 nm with a microtiter ELISA plate reader. Hemolytic rates of zero and 100% were determined in a PBS (35 mM phosphate buffer/150 mM NaCl, pH 7.4) alone and 0.1% Triton X-100, respectively. The percentage of hemolysis was calculated by using the following equation: Percentage hemolysis = [(Abs414 nm in the peptide solution – Abs414 nm in a PBS) / (Abs414 nm in 0.1% Triton X-100 – Abs414 nm in a PBS)] × 100 [25].

### 3.7. Measurement of plasma membrane fluorescence intensity

The anisotropy of the fluorescence from the plasma membrane of exponential *C. albicans* cells labeled with 1,6-diphenyl-1,3,5-hexatriene (DPH; Molecular Probes, Eugene, OR, USA) were detected to investigate changes in membrane dynamics. The cells (1 × 108 /mL), containing (+)-pinoresinol or amphotericin B used as positive control were incubated at a physiological temperature of 28 °C on a rotary shaker at 140 rpm for 2 h. Control cells were incubated without adding compound. For DPH labeling, the pellets were thawed and resuspended in a PBS buffer. The suspension was incubated at 28 °C for 45 min in the presence of 0.6 mM of DPH, followed by several washings in a PBS buffer. The steady-state fluorescence anisotropy was measured using a RF-5301PC spectrofluorometer (Shimadzu, Kyoto, Japan) at 350 nm excitation and 425 nm emission wavelengths [26].

### 3.8. Preparation and microscopic observation of GUVs

Phospholipid giant unilamellar vesicles (GUVs) were prepared by using ITO (indium tin oxide) glasses. A phospholipid mixture solution of PC/1,2-dioleoyl-*sn*-glycero-3-phosphoethanolamine-*N*-(lissamine rhodamine B sulfonyl) (ammonium salt) (18:1 Liss Rhod PE)/PI/ergosterol (5:4:1:2, w/w/w/w) was prepared at a concentration of 3.75 mg/mL in chloroform [18]. The lipid solutions (100–200 μL) were deposited in a spin coater (Spin Coater, ACE-1020 Series) and the glass was coated for 2 min, and then the coated ITO glass was evaporated under a vacuum for 2 h. The following procedure was used in succession; a square frame was created from silicon served as a thickness (2 mm) spacer between the lipid-coated glass and normal glass. The chamber was filled with 10 mM HEPES buffer (pH 7.2) through a hole in the silicon spacer. Immediately, the application of 1.7 V (peak-to-peak, sine wave) and 10 Hz to the ITO electrodes was made by using a sweep function generator (Protek, SWEEP FUNCTION GENERATOR 9205C) for 2 h at room temperature. GUVs from the ITO glass were then detached in conditions of 4 V pp (peak-to-peak) and at 4 Hz for 10 min [15,27]. Ten microliters of GUV were place on an inverted flurorescence phase-contrast microscope (Leica, DFC 420C), and (+)-pinoresinol solutions were treated after the selection of a single GUV.

## 4. Conclusions

The novel antifungal properties of (+)-pinoresinol were investigated. (+)-Pinoresinol exhibited antifungal activities on human pathogenic fungi with no hemolytic effects against human erythrocytes. Although the exact mechanisms of action of (+)-pinoresinol have not been completely elucidated, this study suggests that the compound may act by depolarization or formation of pores in the fungal bilayer membrane. Therefore, it can be concluded that (+)-pinoresinol has considerable fungicidal effects, deserving further investigation for clinical applications.

## Figures and Tables

**Table 1:** Antifungal activity of (+)-pinoresinol.

Compound	MIC (µg/mL)		
	*C. albicans*	*M. furfur*	*T. beigelii*
**(+)-Pinoresinol**	12.5	25	25
**Amphotericin B**	6.25	6.25	6.25

**Table 2:** The concentration of trehalose and glucose caused by (+)-pinoresinol.

Compound	Amounts of trehalose and glucose concentrations (μg/mg)	
	Intracellular glucose and trehalose	Released glucose and trehalose
**Control**	1.89	6.91
**(+)-Pinoresinol**	10.83	11.32
**Amphotericin B**	5.70	20.79

**Table 3:** Hemolytic activity of (+)-pinoresinol against human erythrocytes.

	% Hemolysis (µg/mL)					
	100	50	25	12.5	6.25	3.125
**(+)-Pinoresinol**	0	0	0	0	0	0
**Amphotericin B**	95.33	82.85	63.38	51.51	36.89	25.18
